# α‐Linolenic Acid Alleviated Intestinal Inflammation and Dyshomeostasis Induced by Obesity in Mice

**DOI:** 10.1002/fsn3.71680

**Published:** 2026-03-23

**Authors:** Ying Zhang, Yanxiang Li, Yue Wu, Ziyuan Wang, Jie Liu, Xiao Guan

**Affiliations:** ^1^ School of Health Science and Engineering University of Shanghai for Science and Technology Shanghai China; ^2^ National Grain Industry (Urban Grain and Oil Security) Technology Innovation Center Shanghai China; ^3^ Key Laboratory of Geriatric Nutrition and Health, Ministry of Education Beijing Technology and Business University Beijing China

**Keywords:** α‐linolenic acid, intestinal dyshomeostasis, intestinal inflammation, obesity, TLR4/NFκB signaling pathway

## Abstract

Obesity and its associated metabolic disorders are closely linked to high‐fat diet (HFD)‐induced intestinal inflammation. In this study, we used an HFD‐induced obese mice model to evaluate the regulatory effects of low‐dose, medium‐dose (M‐LA), and high‐dose of α‐linolenic acid (ALA) on intestinal inflammation and dyshomeostasis. After ALA intervention, all dose groups significantly reduced body weight. Especially M‐LA significantly improved serum/hepatic lipid profiles and intestinal histopathology. Furthermore, M‐LA significantly upregulated the expression of tight junction proteins and mucin 2, restored goblet cell numbers, and rebalanced the TH17/Treg cell ratio. M‐LA decreased the Firmicutes/Bacteroidetes ratio in HFD‐fed mice. Additionally, it attenuated hyperproliferation of intestinal stem cells and promoted their differentiation into enteroendocrine cells and goblet cells. We verified its effects on the TLR4/NFκB pathway in both obese mice and LPS‐induced Caco‐2 cells. The intervention effect of ALA was dose‐dependent, with the M‐LA (0.221 g/kg) showing the best improvement effect. This study provides a theoretical foundation for dietary active lipids in obesity regulation and the development of ALA‐based functional foods.

## Graphical abstract

α‐linolenic acid (ALA) could modulate intestinal immune balance and microbiota composition. ALA alleviated intestinal inflammation by suppressing the TLR4/NF‐κB signaling pathway.

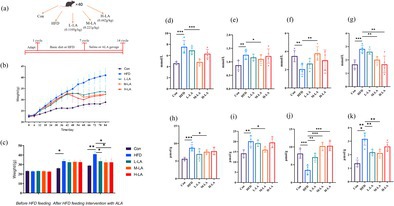

## 1 Introduction

Chronic consumption of a high‐fat diet (HFD) represents one of the primary etiological factors for obesity development. HFD promotes metabolic syndrome progression by inducing systemic chronic low‐grade inflammation, exacerbating oxidative stress, and disrupting lipid metabolism (Chervet et al. 2025; Zhang et al. 2024). Obesity‐associated inflammation induced by long‐term HFD exposure is frequently accompanied by intestinal barrier dysfunction, microbial disorder, and disruption of intestinal epithelial homeostasis. The gut barrier, serving as the first line of defense against pathogen and toxin invasion, maintains its integrity through coordinated actions of tight junction proteins (e.g., Occludin and Claudin‐1), the mucus layer (primarily composed of MUC2 secreted by goblet cells), and immune cell functions (Chen et al. 2025; Cai et al. 2024; Du et al. 2025). Current evidence demonstrates that HFD feeding triggers intestinal inflammation through activation of the TLR4/NF‐κB pathway, leading to tight junction disruption, reduction in goblet cell numbers, and microbial imbalance (Chen et al. 2025; Cai et al. 2024). Furthermore, HFD has been shown to induce abnormal proliferation and differentiation of intestinal stem cells via Wnt/β‐catenin and NOTCH signaling pathways. Such dysregulation of intestinal stem cell may further aggravates intestinal dysfunction (Tian et al. 2015).

In recent years, dietary active lipids have garnered significant attention for their potential roles in modulating inflammatory responses, improving lipid metabolism, and alleviating oxidative stress. Particularly, polyunsaturated fatty acids (PUFAs), including n‐3 series fatty acids such as eicosapentaenoic acid (EPA) and α‐linolenic acid (ALA), have demonstrated remarkable anti‐inflammatory and antioxidant properties (Du et al. 2025; Cao et al. 2024; Bork et al. 2024). However, the specific regulatory effects of different dietary lipids including ALA, conjugated linoleic acid (CLA), and oleic acid (OA) on HFD‐induced obesity, metabolic disorders, and intestinal inflammation remain incompletely understood. Our preliminary studies identified ALA as a potent active lipid for alleviating obesity‐associated metabolic dysregulation using an HFD‐induced obese mice model. The results revealed that ALA not only significantly improved body weight gain, abnormal lipid profiles, and oxidative stress levels in obese mice, but also exhibited potential regulatory effects on gut homeostasis by preserving intestinal tissue morphology and suppressing inflammatory factors expression. Nevertheless, the precise mechanism through which ALA maintained intestinal homeostasis‐ particularly via modulation of gut barrier integrity, microbial composition, and stem cell proliferation/differentiation remains to be fully elucidated.

The TLR4/NFκB signaling pathway serves as a key hub connecting obesity, intestinal inflammation, and metabolic disorders. Pathway activation promotes the release of pro‐inflammatory cytokines (including TNF‐α and IL‐1β) and impairs intestinal epithelial barrier function (Yu et al. 2022; Peng et al. 2025). Current evidence demonstrates that HFD‐induced obesity activates TLR4 receptors through both free fatty acids (FFAs) and gut microbiota‐derived LPS, triggering MyD88‐dependent NFκB nuclear translocation and establishing a vicious cycle of inflammation and oxidative stress (Peng et al. 2025; Wang et al. 2025; Wu et al. 2024). Notably, while n‐3 polyunsaturated fatty acids such as EPA have been shown to alleviate inflammation through TLR4/NFκB pathway inhibition (Bork et al. 2024; Han et al. 2022), whether ALA exerts comparable mechanistic effects remains to be validated.

To systematically investigate the therapeutic potential of ALA, this study established low‐dose, medium‐dose, and high‐dose ALA intervention in HFD‐induced obese mice, with comprehensive evaluation of its effects on intestinal barrier function, microbial composition, and epithelial homeostasis. Our primary aim was to elucidate ALA's multi‐target regulation in alleviating obesity‐associated intestinal inflammation and homeostasis disruption. For mechanistic insights, we integrated in vivo experiments with an in vitro LPS‐induced Caco‐2 cell inflammation model to examine ALA's modulation of key TLR4/NF‐κB pathway factors (TLR4, MyD88, NF‐κB p65, and Traf6) at both transcriptional and protein levels. This multi‐level experimental design provides compelling evidence to determine whether ALA may exert its anti‐inflammatory and barrier‐protective effects through TLR4/NF‐κB pathway regulation, thereby establishing a theoretical foundation for ALA's application as a functional lipid in obesity intervention strategies.

## 2 Materials and Methods

### 2.1 Materials and Reagents

Sixty 6–8‐week‐old male C57BL/6J mice (specific pathogen‐free [SPF] grade) were purchased from Shanghai Jiesijie Laboratory Animal Co. Ltd. (License No.: SCXK[Shanghai]2018‐0004). Experimental materials and reagents were all shown in Table 1. ALA was dissolved in 3% DMSO and then sonicated. The purity of ALA was 99%, and it was extracted from flaxseed oil.

**TABLE 1:** Experimental materials and reagents.

Materials/reagents	Manufacturer/supplier
α‐Linolenic acid (ALA)	Merck Investments (China) Co. Ltd.
Dimethyl sulfoxide (DMSO)	Sangon Biotech (Shanghai) Co. Ltd.
Experimental animal feed	Jiangsu Xietong Medical‐Biological Engineering Co. Ltd.
Total cholesterol (TC) assay kit	Nanjing Jiancheng Bioengineering Institute
Triglyceride (TG) assay kit	Nanjing Jiancheng Bioengineering Institute
Low density lipoprotein cholesterin (LDL‐C) assay kit	Nanjing Jiancheng Bioengineering Institute
High density lipoprotein cholesterin (HDL‐C) assay kit	Nanjing Jiancheng Bioengineering Institute
CatLAse (CAT) assay kit	Beyotime Biotechnology (Shanghai)
Superoxide dismutase (SOD) assay kit	Beyotime Biotechnology (Shanghai)
Glutathione peroxidase (GPx) assay kit	Beyotime Biotechnology (Shanghai)
Malondialdehyde (MDA) assay kit	Beyotime Biotechnology (Shanghai)
Enhanced BCA protein assay kit	Beyotime Biotechnology (Shanghai)
Pre‐stained protein marker (10–180 kDa)	Beyotime Biotechnology (Shanghai)
Western and IP cell lysis buffer	Beyotime Biotechnology (Shanghai)
Western blot antibody dilution buffer	Beyotime Biotechnology (Shanghai)
30% Acr‐Bis solution	Beyotime Biotechnology (Shanghai)
5× SDS‐PAGE loading buffer	Beyotime Biotechnology (Shanghai)
Ultra‐sensitive ECL chemiluminescence kit	Beyotime Biotechnology (Shanghai)
Horseradish peroxidase‐labeled goat anti‐ mouse IgG	Beyotime Biotechnology (Shanghai)
Horseradish peroxidase‐labeled goat anti‐rabbit IgG	Beyotime Biotechnology (Shanghai)
Mouse anti‐β‐Actin antibody	Beyotime Biotechnology (Shanghai)
Tetramethyl ethylenediamine (TEMED)	Shanghai LAddin Biochemical Technology Co. Ltd.
Anchor skim milk powder	Shanghai Vita Chemical Reagent Co. Ltd.
Methanol	Sinopharm Chemical Reagent Co. Ltd. (Shanghai)
Polyvinylidene fluoride (PVDF) membrane	Merck Investments (China) Co. Ltd.
Rabbit polyclonal anti‐TNF‐α antibody	Beyotime Biotechnology (Shanghai)
Mouse monoclonal anti‐IL‐1β antibody	Beyotime Biotechnology (Shanghai)
Rabbit anti‐Claudin‐1 polyclonal antibody	Jiangsu Qinke Biotechnology Research Center Co. Ltd.
Rabbit anti‐Occludin polyclonal antibody	Jiangsu Qinke Biotechnology Research Center Co. Ltd.
DEPC‐treated water	Sangon Biotech (Shanghai) Co. Ltd.
EZ‐10 Spin Column Total RNA Mini‐prep Kit	Sangon Biotech (Shanghai) Co. Ltd.
RT‐qPCR primers	Sangon Biotech (Shanghai) Co. Ltd.
RNase‐free ddH_2_O	Nanjing Vazyme Biotech Co. Ltd.
HiScript III qRT SuperMix for qPCR	Nanjing Vazyme Biotech Co. Ltd.
Taq Pro Universal SYBR qPCR Master Mix	Nanjing Vazyme Biotech Co. Ltd.
Hanks' balanced salt solution (HBSS)	Tianjin Haoyang Biological Products Technology Co. Ltd.
DMEM medium	Thermo Fisher Scientific (USA)
Trypsin–EDTA (0.25%)	Thermo Fisher Scientific (USA)
Non‐essential amino acids (NEAA)	Thermo Fisher Scientific (USA)
Penicillin–streptomycin (100×)	Thermo Fisher Scientific (USA)
Fetal bovine serum (FBS)	Shanghai BasalMedia Technologies Co. Ltd.
Rabbit polyclonal antibody against TLR4	Beyotime Biotechnology (Shanghai)
Mouse monoclonal antibody against MyD88	Thermo Fisher Scientific (USA)
Rabbit polyclonal antibody against TRAF6	Beyotime Biotechnology (Shanghai)
Rabbit polyclonal antibody against NFκB‐p65	Beyotime Biotechnology (Shanghai)

### 2.2 Establishment and Grouping of Experimental Animal Models

Following a 7‐day acclimatization period, mice were randomly divided into six groups (*n* = 8 per group): Normal control group (Con), High‐fat diet‐induced obesity group (HFD), Low‐dose α‐linolenic acid group (L‐LA, 0.1105 g/kg), Medium‐dose α‐linolenic acid group (M‐LA, 0.221 g/kg), High‐dose α‐linolenic acid group (H‐LA, 0.442 g/kg). The different doses of ALA for mice were according to Chinese National Health Standard of WS/T 578.1‐2017. The obesity model was established with 6 days per week for a total of 14 weeks, during which all groups except Con were fed a purified high‐fat feed containing 60% fat supplied. Starting from 7th week, ALA intervention groups received daily oral gavage of their respective doses, while Con and HFD groups received equal volumes of saline, continuing for 14th week. Body weights were recorded weekly throughout the modeling and intervention periods.

At the end of the 14th week, after 16‐h fasting (water allowed), all mice were euthanized for sample collection. All animal procedures strictly adhered to Chinese Laboratory Animal Guidelines and internationally recognized protocols, with prior approval from the Institutional Animal Care and Use Committee (Approval No.: IRB‐AF63‐V1.0).

### 2.3 Determination of Hepatic and Serum Lipid Profiles

Serum and liver tissue samples were collected from all experimental groups. According to the manufacturers' protocols of commercial assay kits, lipid parameters including total cholesterol (TC), triglycerides (TG), low‐density lipoprotein cholesterin (LDL‐C), and high‐density lipoprotein cholesterin (HDL‐C) were quantitatively measured in both serum and liver homogenates.

### 2.4 Histological and Immunohistochemical Analyses of Intestinal Tissues

Jejunal tissues from all experimental groups were processed for hematoxylin and eosin (H&E) staining according to standard protocols (Zhang et al. 2025a, 2025b). Morphometric analysis of villus height and crypt depth was performed using ImageJ software (NIH, USA) to determine villus height‐to‐crypt depth (V/C) ratios.

Goblet cells in jejunal sections were identified by Alcian blue (A&B) staining. Quantification of goblet cells per villus‐crypt unit was conducted using ImageJ software.

Cell proliferation in intestinal crypts was assessed by immunostaining for Ki67, a marker of proliferating cells. The number of Ki67‐positive cells per crypt was quantified using ImageJ software.

### 2.5 Analysis of Gene Expression in Intestinal Tissues and Cells

The mRNA expression levels of inflammatory cytokines in jejunal tissues were determined by qRT‐PCR. Primer stocks were initially diluted to 100 μM using TE buffer, followed by working dilution to 10 μM with DEPC‐treated water and storage at −20°C until use.

Cellular inflammation models were generated in 6‐well plates by incubating cells with 1.5 mL of incomplete medium containing optimized LPS concentrations (10 μg/mL) and 500 μL cell suspension for 24 h. Following successful establishment of the model, the medium was aspirated and cells were washed twice with Hanks' balanced salt solution (HBSS). Subsequently, 2 mL of ALA solution (60 μM) was added for 12 h incubation. After treatment, cells were lysed using IP cell lysis buffer for subsequent RNA isolation. qRT‐PCR was performed using the PrimeScript RT reagent kit and SYBR Premix Ex Taq II, with primer sequences listed in Table 2.

**TABLE 2:** Primer sequences for quantitative real‐time PCR.

Gene	Forward primer (5′–3′)	Reverse primer (3′–5′)
TNF‐α	CAGGCGGTGCCTATGTCTC	CGATCACCCCGAAGTTCAGTAG
IL‐1β	CCCAAGCAATACCCAAAGAA	GCTTGTGCTCTGCTTGTGAG
Occludin	ATCACTTTTCCTGCGGTGAC	GGAACGTGGCCGATATAATG
Claudin‐1	TGTTTTCCCGATGACCTTTC	CAACAACAGGGTTAGCAGCA
MUC2	ATGCCCACCTCCTCAAAGAC	GTAGTTTCCGTTGGAACAGTGAA
ZO‐1	ACCACCAACCCGAGAAGAC	CAGGAGTCATGGACACACA
SOD1	TGGTGGTCCATGAGAAACAA	GTTTACTGCGCAATCCCAAT
SOD2	CTGTCTTCAGCCACACCAGA	CTGCTCTTCCAAAGGTCCTG
SOD3	TCTTCCTGTCCCCATAGCAC	GGTGAGGGTGTCAGAGTGGT
CAT	GCACTGTTCAGCACCTTTGA	GCAAAGGCAGCGTAGGTAAC
GPx‐1	CACGTGATCTCAGCACCATC	AGAAGGCATACACGGTGGAC
GPx‐2	CAGCTTCCAGACCATCAACA	CACTGAGCCCTGAGGAAGAC
Lgr5	CAAGGACGTGAGCATGTATCC	GTAACCACCGTAGTCCGGGTA
Lyz1	GAGACCGAAGCACCGACTATG	CGGTTTTGACATTGTGTTCGC
CDX1	GGACGCCCTACGAATGGATG	GTACCGGCTGTAGTGAACTC
ChgA	ATCCTCTCTATCCTGCGACAC	GGGCTCTGGTTCTCAAACACT
β‐Actin	GATCTGGCACCACACCTTCT	GGGGTGTTGAAGGTCTCAAA
TLR4	AGTTGATCTACCAAGCCTTGAGT	GCTGGTTGTCCCAAAATCACTTT
Myd88	GGCTGCTCTCAACATGCGA	CTGTGTCCGCACGTTCAAGA
NFκB1	AACAGAGAGGATTTCGTTTCCG	TTTGACCTGAGGGTAAGACTTCT
Traf6	ATGCGGCCATAGGTTCTGC	TCCTCAAGATGTCTCAGTTCCAT
RORγt	GACCCACACCTCACAAATTGA	AGTAGGCCACATTACACTGCT
FOXP3	CCCATCCCCAGGAGTCTTG	ACCATGACTAGGGGCACTGTA

### 2.6 Western Blot Analysis of Intestinal Tissue‐Related Proteins and Key Inflammatory Pathway Proteins in Cells

The protein expression levels of Occludin, Claudin‐1, IL‐1β, and TNF‐α in the jejunum tissues of mice were determined by western blot. Additionally, the key protein levels of the TLR4/NF‐κB signaling pathway (TLR4, MyD88, NF‐κB p65, and Traf6) in LPS‐induced Caco‐2 cells were analyzed using western blot (Li et al. 2024).

### 2.7 Measurement of Antioxidant Protein Levels in Intestinal Tissues

After collecting small intestinal tissue samples from mice in each group, the oxidative stress levels were measured and analyzed according to the manufacturer's instructions. Specific indicators included the activities of SOD, CAT, and GPx, as well as the concentration of MDA.

### 2.8 Extraction, Sequencing, and Bioinformatics Analysis of Gut Microbial Total DNA

The cecum contents from mice in each group were collected, and total genomic DNA of the microbial community was extracted. The quality of the genomic DNA was assessed by 1% agarose gel electrophoresis, while concentration and purity were measured using a Nanodrop 2000 spectrophotometer.

The extracted DNA was used as a template to amplify the V3‐V4 hypervariable region of the 16S rRNA gene via PCR with barcoded primers 338F (5′‐ACTCCTACGGGAGGCAGCAG‐3′) and 806R (5′‐GGACTACHVGGGTWTCTAAT‐3′). The PCR products were stored at 4°C. The PCR reaction mixture (20 μL total volume) contained: 4 μL of 5 × TransStart FastPfu buffer, 2 μL of 2.5 mM dNTPs, 0.8 μL each of forward and reverse primers (5 μM), 0.4 μL of TransStart FastPfu DNA polymerase, 10 ng of template DNA, and ddH_2_O to adjust the volume. The PCR amplicons were separated by 2% agarose gel electrophoresis, and target bands were purified using a DNA gel extraction kit. The purified DNA fragments were quantified using a Qubit 4.0 fluorometer. After library preparation with the Rapid DNA‐Seq kit, paired‐end sequencing (PE300) was performed on the Illumina platform (Shanghai Majorbio Bio‐Pharm Technology Co. Ltd.). Subsequent data processing and bioinformatics analysis were conducted via the Majorbio Cloud Platform (https://cloud.majorbio.com).

### 2.9 Establishment of LPS‐Induced Inflammatory Model in Caco‐2 Cells

A 2 mg/mL LPS stock solution was freshly diluted with incomplete DEME medium to gradient concentrations of 0, 2.5, 5, 10, 20, and 40 μg/mL. In 96‐well cell culture plates, each well was inoculated with 1 × 10^5^ Caco‐2 cells in 100 μL complete medium, with six replicate wells per group. To maintain humidity, the peripheral wells were filled with an equal volume of Hanks' balanced salt solution. After 24 h incubation at 37°C with 5% CO_2_ to allow cell adhesion, the culture medium was replaced with LPS solutions at different concentrations for another 24 h treatment. Subsequently, 10 μL of CCK‐8 reagent was added to each well, followed by 1 h incubation. The absorbance at 450 nm was measured using a microplate reader. The optimal LPS concentration for effectively establishing the Caco‐2 cell inflammatory model was determined by analyzing cell viability (Li et al. 2023).

### 2.10 Statistical Analysis

All experiments were repeated at least three times. Data are presented as mean ± standard deviation (SD) and were plotted using GraphPad Prism 9.5.0. Statistical comparisons were performed using either independent samples *t*‐tests or one‐way analysis of variance (ANOVA) followed by Tukey's post hoc test in IBM SPSS Statistics 25. Significant differences were defined as *p < 0.05, **p < 0.01, and ***p < 0.001.

## 3 Results and Discussion

### 3.1 Effects of α‐Linolenic Acid (ALA) on General Characteristics and Lipid Profiles in HFD‐Fed Mice

**FIGURE 1:**
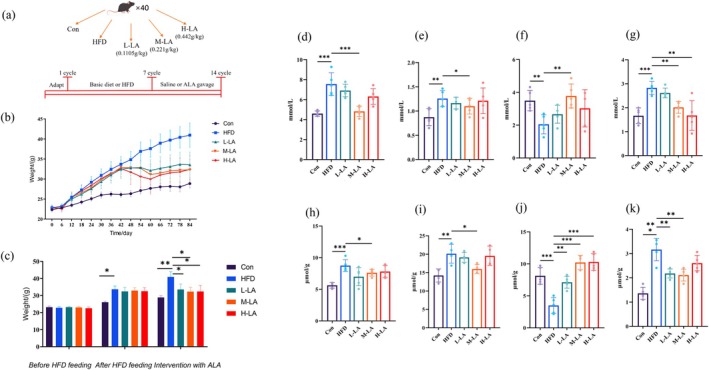
Effects of ALA on body weight and serum and hepatic lipid profiles in HFD‐fed mice. (a) Grouping and intervention protocols of mice, (b) Body weight change trends in mice, (c) Body weights of mice at different stages (different letters in the figure indicate statistically significant differences at *p* ≤ 0.05), (d) Serum total cholesterol (TC) content, (e) Serum triglyceride (TG) content, (f) Serum low‐density lipoprotein cholesterol (LDL‐C) content, (g) Serum high‐density lipoprotein cholesterol (HDL‐C) content, (h) Hepatic TC content, (i) Hepatic TG content, (j) Hepatic LDL‐C content, (k) Hepatic HDL‐C content (*p < 0.05, **p < 0.01, ***p < 0.001).

To investigate the dose‐dependent effects of LA in HFD‐fed mice, three oral gavage doses (low: 0.1105, medium: 0.221, high: 0.442 g/kg) were administered. As shown in Figure 1a, after acclimatization, mice were randomly divided into five groups: a control group (Con) and four HFD groups for obesity induction over six cycles. Following successful model establishment, three experimental groups (L‐LA, M‐LA, H‐LA) received ALA intervention (HFD + ALA) for seven cycles, while the HFD group received saline.

Figure 1b,c demonstrated body weight changes during the experiment. Compared with the Con group, HFD feeding for six cycles significantly increased body weight in model groups. ALA administration markedly suppressed weight gain, whereas the saline‐treated HFD group exhibited sustained weight increase, indicating ALA's mitigating effect on HFD‐induced obesity. Organ weights (Table S1) followed similar trends to body weight changes. Notably, the M‐LA group showed organ weights (heart, liver, spleen, kidney) closest to the Con group. These results suggest that ALA effectively attenuates HFD‐induced increases in body and organ weights, with medium‐dose ALA exhibiting the most pronounced organ weight normalization. However, in terms of body weight, no significant differences were observed among these three ALA doses. Therefore, further experiments are needed to explore the changes in the effects of ALA intake in mice.

Serum and hepatic levels of TC, TG, HDL‐C, and LDL‐C are key indicators of lipid metabolism. In this study, we measured these lipid parameters in both serum (Figure 1d–g) and liver (Figure 1h–k) tissues to evaluate the effects of ALA. The results demonstrated that ALA alleviated HFD‐induced dyslipidemia in mice. Compared with the HFD group, the M‐LA group exhibited significant improvements in abnormal TC, TG, HDL‐C, and LDL‐C levels. Although L‐LA showed a modest mitigating effect, no statistically significant difference was observed relative to the HFD group. Meanwhile, H‐LA significantly improved serum LDL‐C and hepatic HDL‐C levels, but its effects on other lipid parameters were less pronounced than those of M‐LA and did not reach statistical significance compared with the HFD group. Our experiment aims to investigate the best effect of these three concentrations of ALA in alleviating obesity in mice, and the data showed that there was no linear dependence between effect and dose of ALA. The specific mechanism of the relationship between low, medium, high doses of ALA and their effects needs further exploration. Thus, among the different ALA doses tested, medium‐dose ALA exerted the most effective intervention in ameliorating HFD‐induced lipid metabolic disorders.

### 3.2 Effects of ALA on Intestinal Inflammatory Factors and Oxidative Stress in HFD‐Fed Mice

Extensive studies have demonstrated that long‐term HFD feeding leads to excessive fat accumulation and adipocyte hypertrophy in mice, triggering the release of pro‐inflammatory cytokines such as IL‐1β and TNF‐α from adipose tissue. Additionally, increased free fatty acid release activates inflammatory pathways (e.g., NF‐κB and TLR4), promoting systemic inflammation. HFD‐induced gut microbiota dysbiosis further elevates intestinal lipopolysaccharide (LPS) production. When systemic inflammation disrupts the intestinal barrier, LPS enters circulation, exacerbating metabolic dysfunction and obesity (Tilg et al. 2020). Therefore, mitigating inflammation and breaking the vicious cycle between inflammation and obesity are crucial for alleviating HFD‐induced metabolic disorders.

**FIGURE 2:**
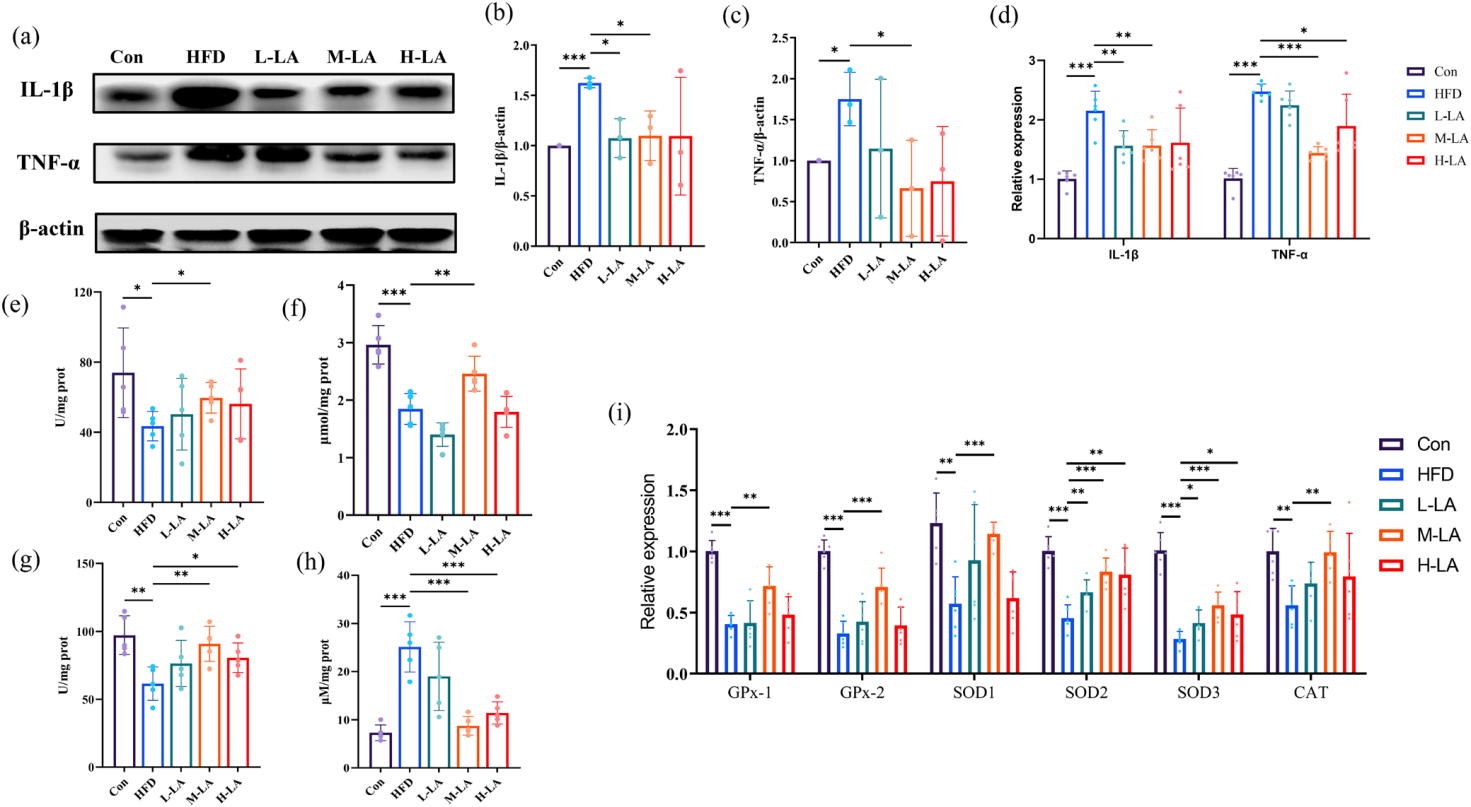
Effects of ALA on intestinal inflammation and oxidative stress in HFD‐fed mice. (a) Western blot analysis of inflammatory factors in small intestinal tissue, (b) Quantitative results of IL‐1β protein expression, (c) Quantitative results of TNF‐α protein expression, (d) mRNA expression levels of inflammatory‐related genes in small intestinal tissue, (e) Superoxide dismutase (SOD) activity, (f) Catalase (CAT) activity, (g) Glutathione peroxidase (GPx) activity, (h) Malondialdehyde (MDA) content, (i) mRNA expression levels of oxidative stress‐related genes (*p < 0.05, **p < 0.01, ***p < 0.001).

In this study, we assessed the regulatory effects of different ALA doses on jejunal inflammation using western blot and qPCR. As shown in Figure 2a–d, 6 weeks of HFD feeding significantly increased intestinal IL‐1β and TNF‐α levels compared to the Con group. However, ALA intervention reduced these pro‐inflammatory markers to varying degrees, with the M‐LA group exhibiting the most significant suppression compared to the HFD group. Although L‐LA and H‐LA also showed anti‐inflammatory effects, their efficacy was less pronounced than that of M‐LA. Both protein and gene analyses yielded consistent results, confirming that ALA effectively suppresses HFD‐induced intestinal inflammation, with M‐LA demonstrating the optimal inhibitory effect. These findings further support the anti‐inflammatory benefits of ALA in obesity management.

Since HFD‐induced obesity and intestinal inflammation exacerbate oxidative stress, creating a vicious cycle among obesity, inflammation, and oxidative stress, we measured oxidative stress markers in jejunal tissues using commercial assay kits and qPCR. Specifically, we evaluated the activities of antioxidant enzymes (SOD, CAT, GPx) and malondialdehyde (MDA) content to assess intestinal oxidative stress levels.

As shown in Figure 2e–i, compared with the Con group, HFD‐fed mice exhibited significantly decreased SOD, CAT, and GPx activities along with increased MDA content in intestinal tissues. M‐LA intervention remarkably reversed these HFD‐induced alterations by enhancing antioxidant enzyme activities and reducing MDA levels. Although H‐LA showed beneficial effects on GPx activity and MDA content, it failed to ameliorate the impaired SOD and CAT activities. Consistent with these findings, mRNA expression analysis revealed that HFD feeding significantly downregulated oxidative stress‐related genes (SOD1, SOD2, SOD3, CAT, GPx‐1, and GPx‐2) compared to the Con group. All ALA treatment groups attenuated these changes, with the M‐LA group demonstrating the most pronounced effects and showing significant differences versus the HFD group across all measured parameters. These results collectively indicated that medium‐dose LA provides optimal protection against HFD‐induced intestinal oxidative stress.

### 3.3 Effects of ALA on Intestinal Histological Morphology and Mucin Layer‐Related Factors in HFD‐Fed Mice

**FIGURE 3:**
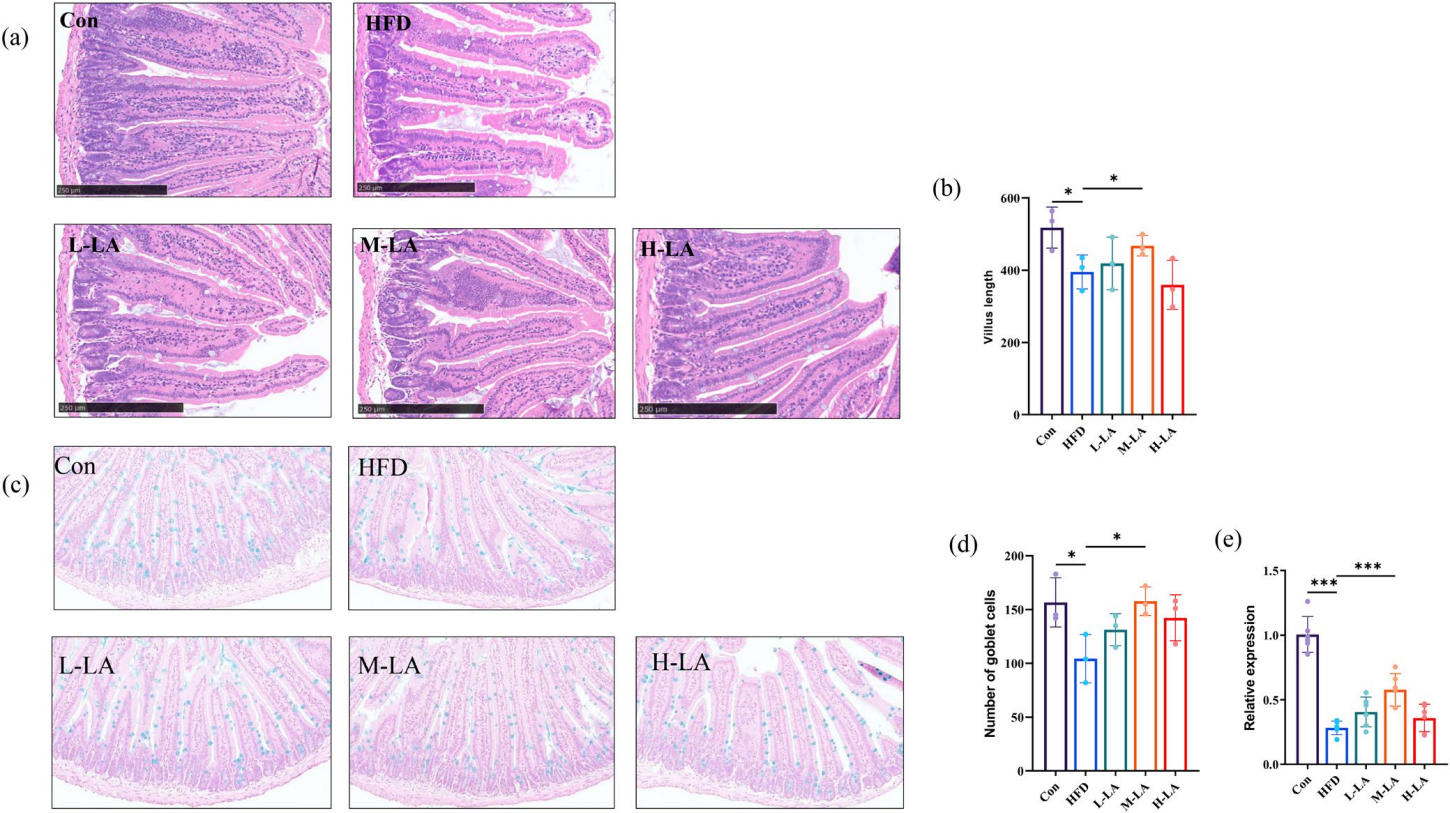
Effects of ALA on intestinal morphology and mucus layer parameters in HFD‐fed mice. (a) Representative H&E staining of small intestinal tissue, (b) Quantitative analysis of small intestinal villus length, (a) A&B staining of small intestinal tissue, (b) Quantitative analysis of goblet cell numbers, (c) Relative mRNA expression of MUC2 mucin (*p < 0.05, **p < 0.01, ***p < 0.001).

H&E staining of small intestinal tissue served as one indicator to evaluate morphological damage induced by HFD feeding. As shown in Figure 3a,b, presenting jejunal H&E staining results and villus length measurements, HFD feeding significantly compromised intestinal morphology compared to the Con group, manifesting as shortened villi and disrupted architecture with sparse arrangement. M‐LA intervention effectively ameliorated these alterations, demonstrating significantly increased villus length and improved villus density. While H‐LA enhanced villus compactness, it failed to rescue the HFD‐induced villus shortening. L‐LA showed minimal effects on both villus length and spacing, with no significant difference from the HFD group. These findings collectively indicated that M‐LA provides optimal protection against HFD‐induced intestinal morphological damage, particularly in restoring villus length and maintaining proper epithelial architecture.

Intestinal goblet cells are specialized secretory cells distributed along the intestinal epithelium that produce MUC2, the primary component of the intestinal mucus layer. This mucus layer constitutes a critical element of the intestinal physical barrier. Both goblet cells and MUC2 collectively maintain intestinal barrier function, protecting the gut from pathogens and mechanical damage, thereby playing a vital role in intestinal health (McCauley and Guasch 2015). The number and content of these two serve as an important indicator of intestinal barrier integrity. Numerous studies have demonstrated that intestinal inflammation leads to reduced goblet cell numbers and MUC2 expression, compromising the intestinal physical barrier and overall gut health (Du et al. 2025). In this study, we employed A&B staining and qPCR to assess goblet cell numbers and MUC2 expression in the jejunum.

As shown in Figure 3c–e, HFD‐induced obesity significantly decreased both goblet cell density and MUC2 levels. LA intervention at various doses restored these parameters to varying degrees. Specifically, M‐LA significantly increased goblet cell numbers compared to the HFD group (*p* < 0.05). For MUC2 expression, both L‐LA and M‐LA showed significant restorative effects, with M‐LA demonstrating the most pronounced improvement. These findings indicated that appropriate ALA supplementation can mitigate HFD‐induced damage to the intestinal physical barrier, preserve barrier integrity, and maintain intestinal homeostasis. Among the tested doses, M‐LA exhibited the most favorable protective effects.

### 3.4 Effects of ALA on Intestinal Tight Junction Proteins, Lysozyme, and Immune Cell Markers in HFD‐Fed Mice

Intestinal tight junction proteins, including transmembrane proteins (Claudin‐1, Occludin) and cytoplasmic scaffolding proteins (ZO‐1), are specialized proteins located within epithelial tight junctions. These proteins form continuous “sealing strands” that constitute the intestinal physical barrier, playing crucial roles in maintaining intestinal barrier integrity, immune homeostasis, and metabolic balance. Certain tight junction proteins also participate in regulating cell proliferation, differentiation, and apoptosis (Fan et al. 2022; Neurath et al. 2025). Numerous studies have demonstrated that HFD‐induced obesity and intestinal inflammation disrupt tight junctions, reducing the expression of Claudin‐1, Occludin, and ZO‐1, thereby impairing intestinal barrier function (Chen et al. 2025). In this study, we evaluated the effects of different ALA doses on intestinal barrier function.

**FIGURE 4:**
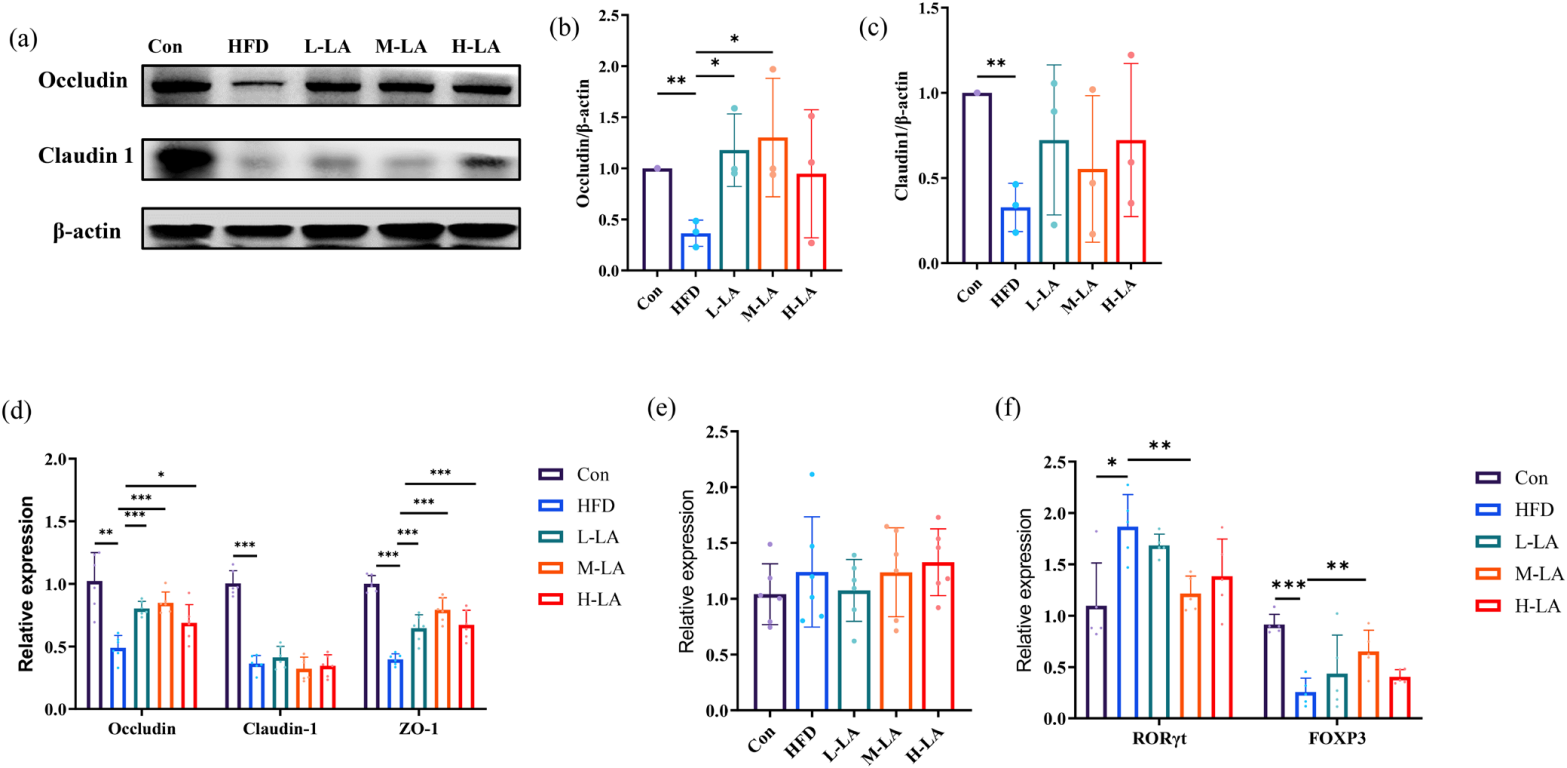
Effects of ALA on intestinal tight junction proteins and lysozyme and immune cells in HFD‐fed mice. (a) Representative western blot images of tight junction proteins, (b) Quantitative analysis of Occludin protein expression, (c) Quantitative analysis of Claudin‐1 protein expression, (d) Relative mRNA expression levels of tight junction proteins associated genes, (e) Relative mRNA expression levels of lysozyme gene, (f) Relative mRNA expression levels of immune cells associated genes (*p < 0.05, **p < 0.01, ***p < 0.001).

As shown in Figure 4a–c, HFD feeding significantly decreased the expression of Occludin and Claudin‐1 in jejunal tissues. Both L‐LA and M‐LA significantly increased Occludin expression, counteracting the HFD‐induced reduction, with M‐LA showing greater efficacy than L‐LA. However, none of the LA doses significantly improved Claudin‐1 expression. Consistent with western blot results, qRT‐PCR analysis (Figure 4d) demonstrated that all ALA doses successfully reversed the HFD‐induced downregulation of ZO‐1 expression, with M‐LA again exhibiting the most pronounced effect. These findings collectively indicated that M‐LA provides optimal protection against HFD‐induced tight junction disruption.

The intestinal chemical barrier is primarily composed of gastric acid, digestive juices, antimicrobial peptides, various enzymes secreted by the gastrointestinal tract, and bile secreted by the liver. Among these components, lysozyme, an alkaline enzyme that hydrolyzes peptidoglycan in bacteria, is a product secreted by Paneth cells. It can directly eliminate pathogenic bacteria and protect intestinal health (De Souza et al. 2019).

As shown in Figure 4e, the effects of different ALA gavage doses on the gene expression of lysozyme in the jejunum of HFD‐fed mice were examined. The results indicated that HFD feeding did not significantly affect the gene expression of intestinal lysozyme in mice. Moreover, ALA intervention at varying doses also showed no notable effect. Therefore, it can be inferred that HFD feeding had no significant impact on Paneth cells in the intestinal crypts or on the secretion of lysozyme by these cells, and ALA intervention did not exert a discernible influence either.

Numerous studies have demonstrated that HFD‐induced obesity leads to intestinal immune dysfunction. Among key immune cell populations, Th17 cells are a pro‐inflammatory T‐cell subset that secretes various inflammatory cytokines, including IL‐6, IL‐17, and TNF‐α (Dardalhon et al. 2008). In contrast, regulatory T cells (Tregs) produce anti‐inflammatory factors and immunosuppressive molecules that inhibit excessive immune responses and maintain immune homeostasis (Cai et al. 2024). An imbalance between Th17 and Treg cells is associated with multiple autoimmune diseases, and restoring their equilibrium has been identified as a potential therapeutic strategy (Cai et al. 2024). In this study, we quantified the effects of different ALA doses on the expression of immune cell signature genes (RORγt for Th17, FOXP3 for Treg) in the jejunum of HFD‐fed mice. As shown in Figure 4f, HFD feeding significantly increased Th17 cell abundance while reducing Treg cell numbers in intestinal tissues. Notably, M‐LA intervention markedly alleviated these effects, suppressing HFD‐induced intestinal hyperimmune responses and restoring immune balance. In contrast, neither L‐LA nor H‐LA produced significant improvements.

### 3.5 Effects of ALA on Gut Microbiota Abundance and Diversity in HFD‐Fed Mice

The gut microbiota refers to the complex microbial community colonizing the gastrointestinal tract, the composition of which is influenced by genetic, dietary, pharmacological, and environmental factors. The balance of gut microbiota is closely associated with host nutritional metabolism, immune regulation, barrier function, and neurological health. Numerous studies have demonstrated that obese individuals exhibit an increased Firmicutes/Bacteroidetes (F/B) ratio in their gut microbiomes. However, this ratio decreases following weight loss or interventions such as dietary modifications and pharmacological treatments (Wang et al. 2023). Additionally, obesity is often accompanied by reduced gut microbial diversity, which may impair normal physiological functions (Turpin et al. 2016). To investigate the impact of ALA on gut microbiota composition, we analyzed the cecal microbial abundance and diversity in HFD‐fed mice using 16S rRNA gene sequencing.

**FIGURE 5:**
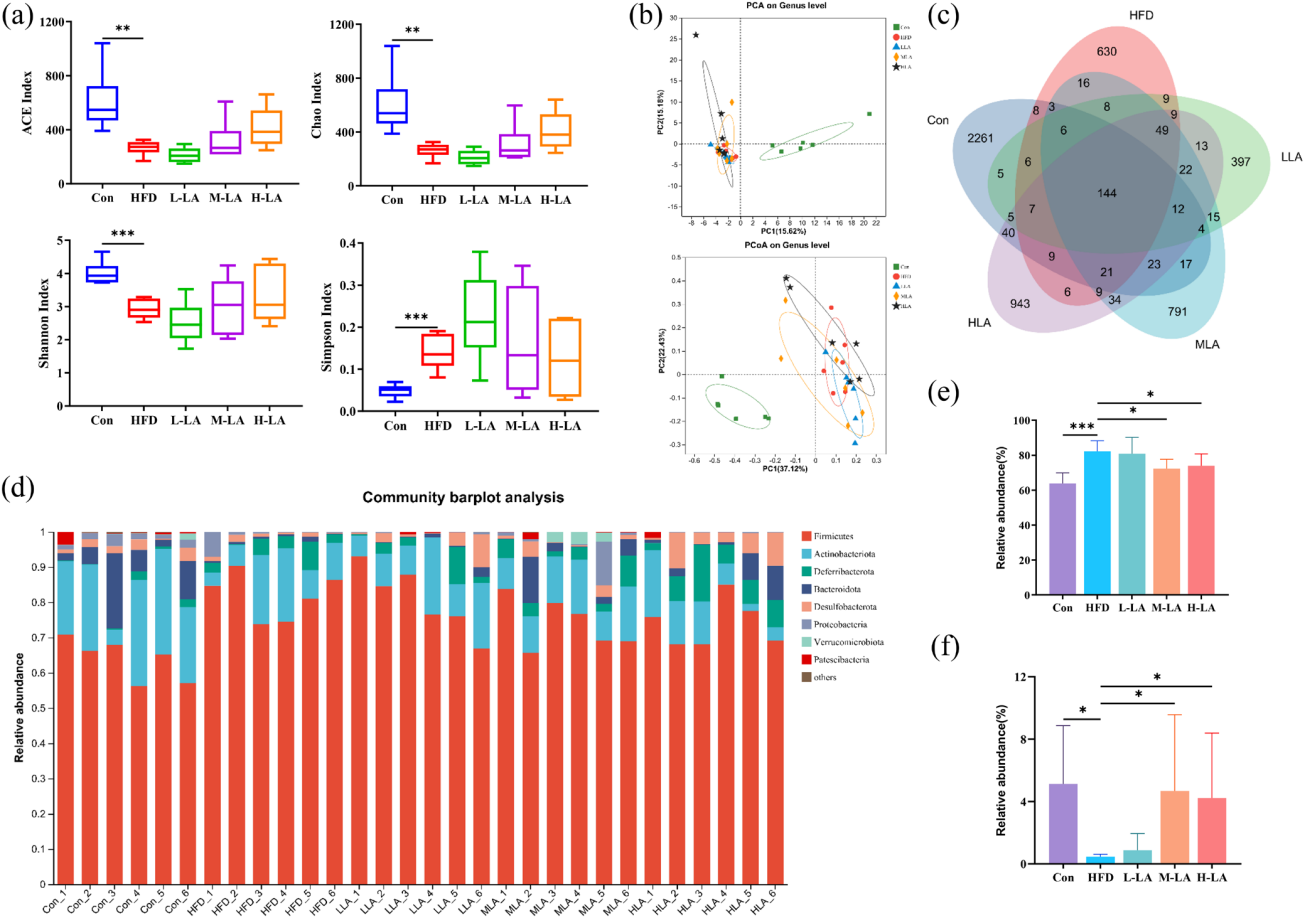
Effects of ALA on gut microbiota abundance and diversity in HFD‐fed mice. (a) α‐Diversity indices (ACE, Chao, Shannon, Simpson) of gut microbiota, (b) β‐diversity analysis (PCA, PCoA) of gut microbiota, (c) Venn diagram of gut microbial composition, (d) Bar plot of microbial community structure, (e) Relative abundance of Firmicutes, (f) Relative abundance of Bacteroidetes (*p < 0.05, **p < 0.01, ***p < 0.001).

Figure 5a–e presented the gut microbiota analysis results. The data demonstrated that HFD feeding significantly reduced microbial diversity indices of ACE, Chao, and Shannon (*p* < 0.01), while increasing the Simpson index, indicating successful induction of obesity‐associated gut microbiota dysbiosis in mice. However, ALA intervention at various doses did not significantly improve microbial diversity in obese mice. Consistent results were observed in PCA/PCoA analyses and Venn diagrams. At the phylum level, HFD feeding markedly increased Firmicutes abundance, decreased Bacteroidetes abundance, and consequently elevated the Firmicutes/Bacteroidetes (F/B) ratio, aligning with previous reports. Notably, M‐LA and H‐LA effectively reversed these trends, reducing the F/B ratio by restoring Bacteroidetes and suppressing Firmicutes proliferation. F/B ratio was different from microbial alpha diversity; alpha diversity reflects the diversity of gut microbiota in the cecum. In contrast, no significant structural changes were observed at the genus level following ALA intervention. In summary, M‐LA and H‐LA supplementation ameliorated HFD‐induced phylum‐level dysbiosis (F/B ratio imbalance) but exerted no measurable effects on overall microbial diversity or genus‐level composition.

### 3.6 Effects of ALA on Intestinal Stem Cell Proliferation and Terminal Differentiation in HFD‐Fed Mice

Intestinal stem cells (ISCs) in mice, located at the base of the crypts, are a type of adult stem cell with self‐renewal and multipotent differentiation capabilities. Through proliferation and differentiation, they continuously replenish intestinal epithelial cells, maintain intestinal barrier integrity, and ensure normal intestinal function. A growing body of evidence suggests that long‐term HFD consumption leads to excessive ISC proliferation, thereby increasing the risk of intestinal carcinogenesis (Deng et al. 2022). Thus, the regulation of ISCs under HFD conditions warrants attention. In this study, we assessed the differential effects of varying doses of ALA on the modulation of jejunal crypt stem cells in HFD‐fed mice using Ki67‐specific immunohistochemistry and qPCR to evaluate the proliferation marker gene Lgr5.

**FIGURE 6:**
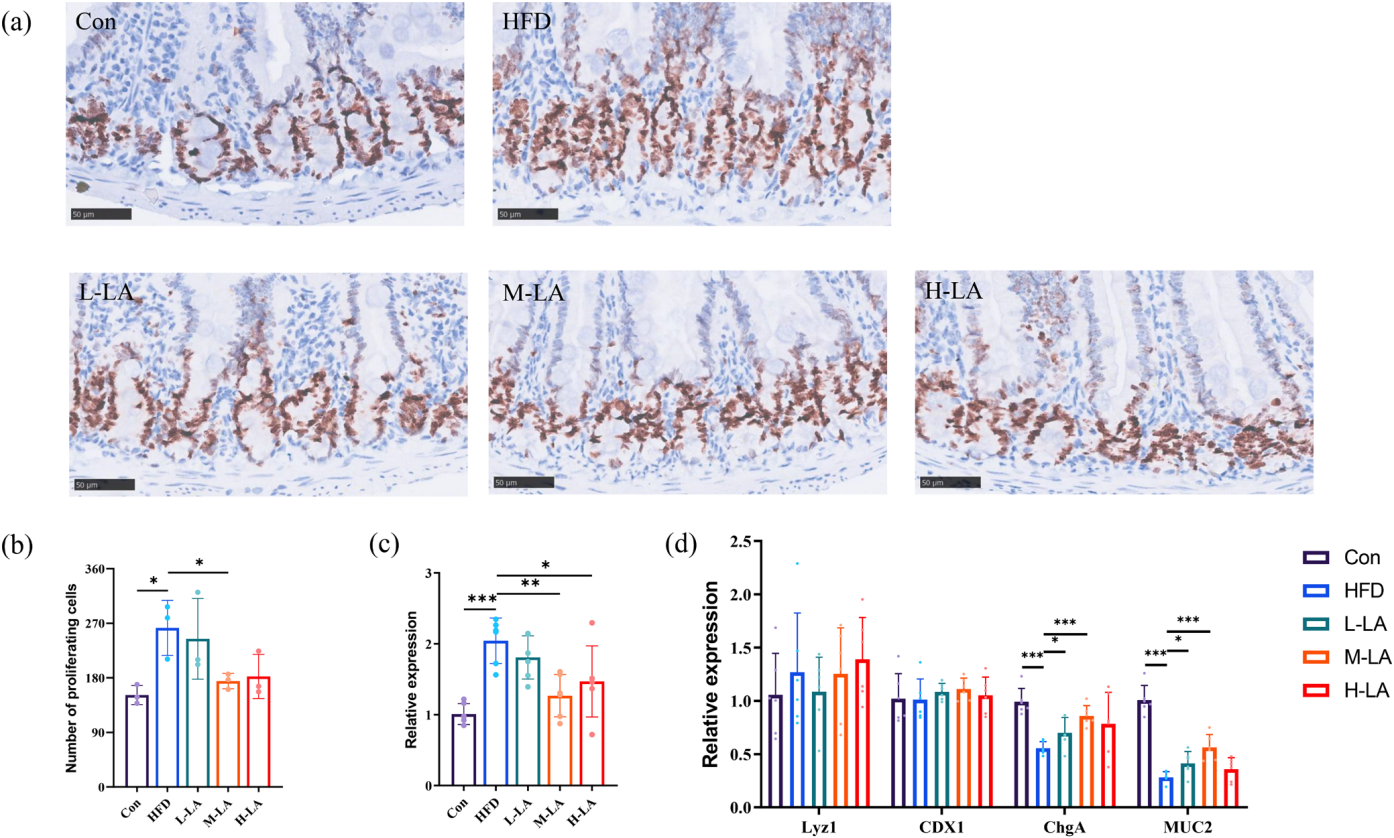
Effects of ALA on small intestinal stem cell proliferation and terminal differentiation in HFD‐fed mice. (a) Representative images of Ki67 staining in small intestinal crypts (scale bar: 50 μm), (b) Quantitative analysis of proliferating cells per crypt, (c) Relative mRNA expression levels of *Lgr5* in intestinal crypts, (d) Relative mRNA expression levels of *Lyz1*, *CDX1*, *ChgA*, *MUC2* (*p < 0.05, **p < 0.01, ***p < 0.001).

As shown in Figure 6a–c, both staining and qPCR results demonstrated that HFD feeding significantly increased the number of proliferating cells in the intestinal crypts compared to the Con group. Notably, the M‐LA group exhibited a marked reversal of HFD‐induced ISC hyperproliferation, indicating that a moderate LA dose could alleviate abnormal ISC proliferation caused by HFD‐induced obesity. In contrast, no significant alleviation was observed in the L‐LA and H‐LA groups. qPCR analysis further revealed that HFD feeding upregulated the expression of Lgr5, a marker gene for proliferating cells, and this trend was significantly attenuated by M‐LA and H‐LA intervention. In summary, M‐LA intervention ameliorated HFD‐induced aberrant crypt stem cell proliferation, while both M‐LA and H‐LA interventions suppressed the overexpression of Lgr5, demonstrating the regulatory role of ALA in mitigating ISC dysfunction.

ISCs maintain intestinal epithelial homeostasis through multipotent differentiation, giving rise to absorptive enterocytes, goblet cells, enteroendocrine cells, and Paneth cells. The continuous renewal of these epithelial lineages ensures critical intestinal functions, including nutrient absorption, waste metabolism, immune protection, and barrier integrity. Studies have demonstrated that HFD‐induced ISC impairment may dysregulate the Wnt and Notch signaling pathways, which govern terminal differentiation, thereby disrupting epithelial homeostasis and compromising intestinal health (Tian et al. 2015). To investigate the effects of varying ALA intake on ISC differentiation, we quantified the expression of specific marker genes in murine intestinal epithelium using qPCR.

As shown in Figure 6d, qPCR analysis revealed that chronic HFD feeding significantly downregulated the expression of enteroendocrine (ChgA) and goblet cell (MUC2) markers compared to the Con group. Supplementation with L‐LA and M‐LA markedly restored their expression, with M‐LA exhibiting the most pronounced efficacy. In contrast, H‐LA showed no significant improvement. Notably, the Paneth cell marker Lyz1 and absorptive enterocyte marker CDX1 remained stable across all groups following HFD and ALA interventions. In summary, prolonged HFD consumption significantly suppressed enteroendocrine and goblet cell gene expression, while M‐LA showed optimal efficacy in alleviating these alterations.

### 3.7 Effects of ALA on LPS‐Induced Inflammation, Barrier Function, and Key Genes Expression in the TLR4/NF‐κB Signaling Pathway in Caco‐2 Cells

**FIGURE 7:**
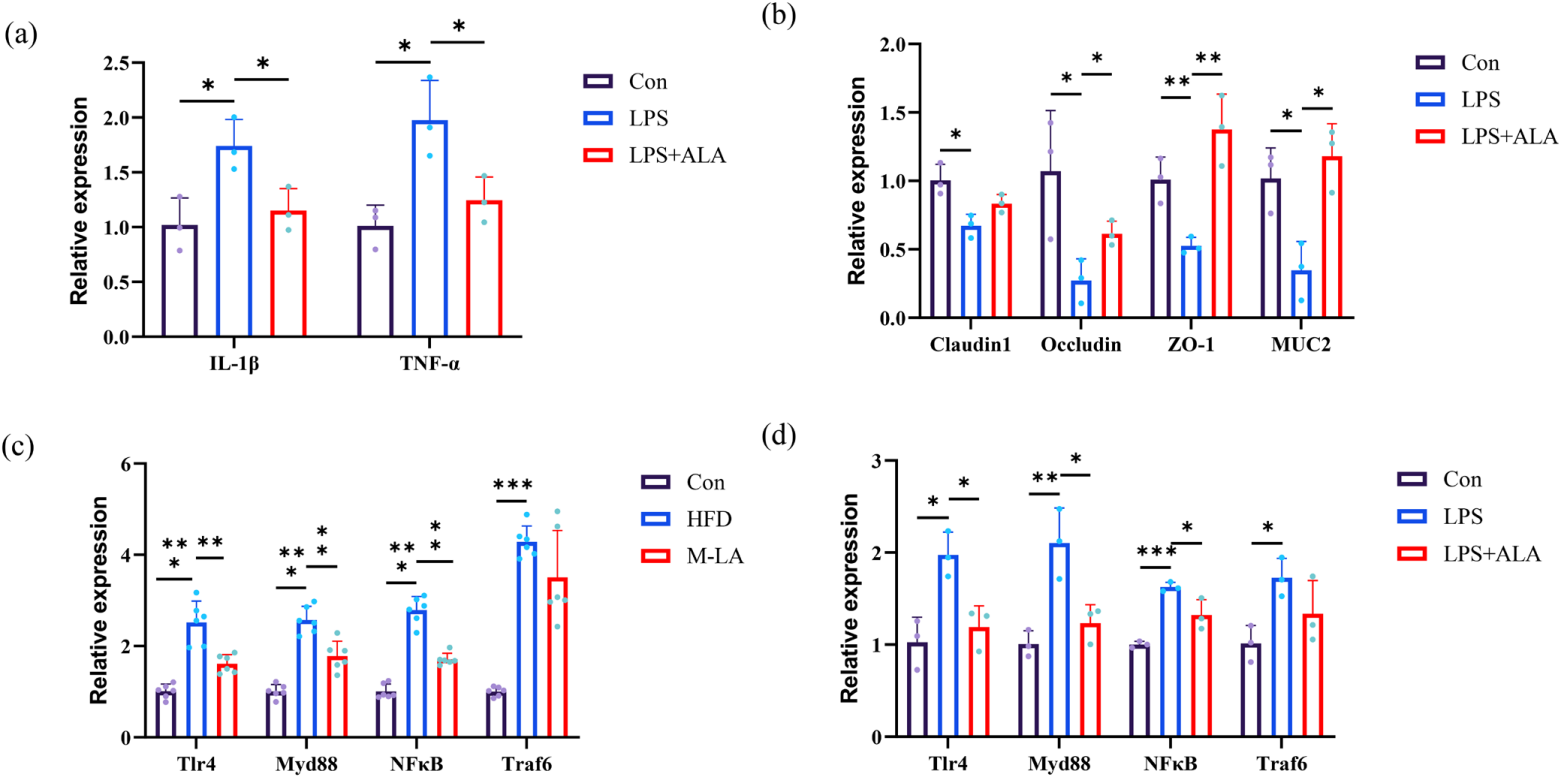
Effects of ALA on LPS induced inflammation, barrier, and key gene expression of TLR4/NF‐κB signaling pathway in Caco‐2 cells. (a) Relative mRNA expression levels of TNF‐α and IL‐1β in Caco‐2 cells, (b) Relative mRNA expression levels of Claudin‐1, Occludin, ZO‐1, MUC2 in Caco‐2 cells, (c) Relative mRNA expression levels of TLR4, MyD88, NF‐κB, and TRAF6 in intestinal tissues of HFD‐fed mice, (d) Relative mRNA expression levels of TLR4, MyD88, NF‐κB, and TRAF6 in Caco‐2 cells (*p < 0.05, **p < 0.01, ***p < 0.001).

To evaluate whether LPS successfully induced an inflammatory and barrier dysfunction model in Caco‐2 cells, thereby simulating intestinal inflammation in vitro, we treated Caco‐2 cells with LPS (10 μg/mL) and ALA (60 μM) (Figure S1). The mRNA expression levels of pro‐inflammatory cytokines TNF‐α and IL‐1β were measured. As shown in Figure 7a, after 24 h of LPS induction, the expression levels of TNF‐α and IL‐1β were significantly elevated. However, following 12 h of ALA incubation, the mRNA levels of these inflammatory cytokines were markedly reduced. These results indicated that ALA treatment for 12 h can effectively attenuate LPS‐induced inflammation, exerting an anti‐inflammatory effect, which aligns with the intestinal anti‐inflammatory effects of LA observed in HFD‐fed mice in our vivo experiments.

Studies have shown that inflammatory cytokines (e.g., TNF‐α, IL‐1β) downregulate the expression of intestinal tight junction proteins, leading to disrupted barrier integrity. Intestinal epithelial cells form a physical and biochemical barrier, segregating host tissues from luminal pathogens to maintain intestinal homeostasis and overall health. Impaired barrier function is a critical factor in intestinal dysregulation (Chen et al. 2025; Du et al. 2025). Previous research has demonstrated that in LPS‐induced Caco‐2 cell inflammation and barrier dysfunction models, the secretion of tight junction proteins (Claudin‐1, Occludin, ZO‐1) and mucin MUC2 is significantly reduced (Liu et al. 2023). As illustrated in Figure 7b, LPS stimulation significantly decreased the expression of Claudin‐1, Occludin, ZO‐1, and MUC2, consistent with existing literature. However, 12 h of ALA incubation notably restored the levels of Occludin, ZO‐1, and MUC2, though it had no significant effect on Claudin‐1. In summary, LA treatment for 12 h ameliorated LPS‐induced barrier disruption in Caco‐2 cells, reinforcing its barrier‐protective role. These findings agree with the barrier‐protective effects of LA observed in HFD‐fed mice.

The TLR4/NF‐κB pathway is a signaling cascade composed of Toll‐like receptor 4 (TLR4) and Nuclear Factor kappa B (NF‐κB). Studies indicated that long‐term HFD‐induced obesity promotes adipose tissue accumulation, adipocyte hypertrophy, and hypoxia, leading to the release of free fatty acids (FFAs) that activate the TLR4/NF‐κB inflammatory pathway, thereby triggering intestinal inflammation (Gupta et al. 2015). Additionally, chronic HFD consumption disrupts the intestinal barrier and induces gut microbiota dysbiosis, allowing pathogen‐derived LPS to translocate into circulation, causing metabolic endotoxemia. This further activates the TLR4/NF‐κB pathway, exacerbating both intestinal and systemic inflammation (Peng et al. 2025). To investigate the possible mechanism by which ALA modulates intestinal inflammation and determine whether it may suppress HFD‐induced inflammation via regulation of the TLR4/NF‐κB pathway, we quantified the mRNA expression levels of key pathway factors (TLR4, MyD88, NF‐κB, and TRAF6). As shown in Figure 7c, HFD feeding significantly upregulated TLR4, MyD88, NF‐κB, and TRAF6 expression in intestinal tissues, confirming activation of the TLR4/NF‐κB pathway. Notably, ALA intervention markedly downregulated TLR4, MyD88, and NF‐κB expression, though it had no significant effect on TRAF6. In conclusion, LA suppressed HFD‐induced activation of the TLR4/NF‐κB pathway by reducing the mRNA expression of TLR4, MyD88, and NF‐κB, thereby attenuating intestinal inflammation.

Current studies demonstrate that LPS induction activates the TLR4/NF‐κB inflammatory signaling pathway in Caco‐2 cells, thereby increasing inflammatory responses (Peng et al. 2025; Liu et al. 2023). To further investigate whether ALA alleviates inflammation through modulation of the TLR4/NF‐κB pathway and elucidates its regulatory mechanism, we quantified mRNA expression levels of key genes in this pathway. As shown in Figure 7d, LPS induction significantly upregulated mRNA expression of TLR4, MyD88, NF‐κB, and TRAF6 in Caco‐2 cells compared to the Con group, confirming activation of the TLR4/NF‐κB inflammatory pathway. Following 12‐h M‐LA incubation of inflamed cells, the expression levels of TLR4, MyD88, and NF‐κB were markedly reduced in Caco‐2 cells. These results indicated that ALA can modulate intestinal inflammation by suppressing the expression of key genes in the TLR4/NF‐κB signaling pathway, consistent with the inhibitory trend observed in intestinal tissues of HFD‐fed mice treated with ALA.

### 3.8 Effects of ALA on Key Protein Expression in the TLR4/NF‐κB Signaling Pathway in LPS‐Induced Caco‐2 Cells

**FIGURE 8:**
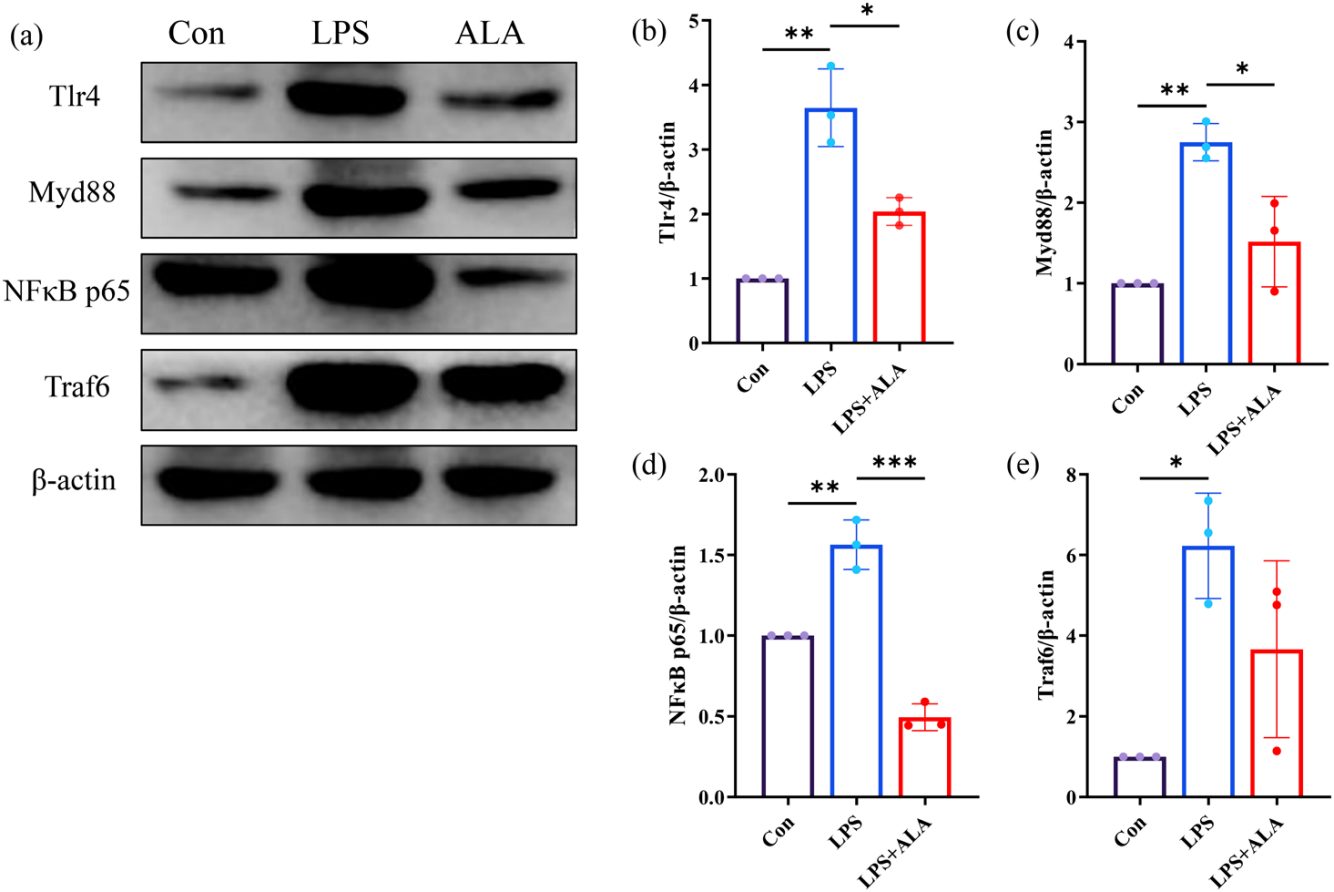
Effects of ALA on key protein expression in the TLR4/NF‐κB signaling pathway in LPS‐induced Caco‐2 cells. (a) Representative western blot images of Caco‐2 cell pathway‐related proteins, (b) Quantitative results of TLR4 protein expression, (c) Quantitative results of Myd88 protein expression, (d) Quantitative results of NF‐κB p65 protein expression, (e) Quantitative results of Traf6 protein expression (*p < 0.05, **p < 0.01, ***p < 0.001).

To further evaluate the inhibitory effects of ALA on the TLR4/NF‐κB inflammatory signaling pathway, we detected the expression levels of key proteins in this pathway. As shown in Figure 8a–e, we examined the protein expression levels of TLR4, MyD88, NF‐κB p65, and TRAF6. The results demonstrated that compared with the Con group, LPS treatment significantly increased the expression levels of all key proteins (TLR4, MyD88, NF‐κB p65, and TRAF6) in the TLR4/NF‐κB signaling pathway. However, ALA intervention showed differential inhibitory effects, significantly reducing the expression levels of TLR4, MyD88, and NF‐κB p65, while exhibiting no significant regulatory effect on TRAF6 protein expression. These findings indicated that ALA intervention can significantly downregulate the expression levels of key proteins (TLR4, MyD88, and NF‐κB p65) in the TLR4/NF‐κB inflammatory signaling pathway, potentially by inhibiting the activation of this pathway and alleviating intestinal inflammation.

## 4 Conclusion

Compared to the HFD group, the M‐LA (0.221 g/kg) group markedly attenuated body weight gain and organ weight increase while reversing abnormal lipid levels in both serum and liver. It also significantly upregulated the expression of tight junction proteins (Occludin and ZO‐1) and the mucin layer protein MUC2. M‐LA and L‐LA intervention markedly reduced the Firmicutes/Bacteroidetes (F/B) ratio, ameliorating microbiota dysbiosis, though no significant effect on microbial alpha diversity was observed. M‐LA maintained intestinal epithelial homeostasis by regulating the proliferation and differentiation of intestinal stem cells.

ALA ameliorated intestinal inflammation and epithelial barrier dysfunction in LPS‐induced Caco‐2 cell model. In vivo experiments demonstrated that HFD feeding significantly upregulated the gene expression levels of TLR4, MyD88, NF‐κB, and Traf6 in mouse intestines, while ALA intervention markedly reduced the expression of TLR4, MyD88, and NF‐κB. In vitro studies further showed that ALA (60 μM) significantly inhibited LPS‐induced protein expression of TLR4, MyD88, and NF‐κB p65 in Caco‐2 cells. These findings collectively indicated that ALA may exert its anti‐inflammatory effects through targeted inhibition of the TLR4/NF‐κB pathway. The mechanism of ALA exerts its anti‐inflammatory and barrier‐protective effects will be further explored.

## Author Contributions

**Yanxiang Li:** writing – review and editing (equal). **Yue Wu:** data curation (equal), project administration (equal). **Ziyuan Wang:** investigation (equal), software (equal). **Jie Liu:** funding acquisition (equal).

## Funding

This work was supported by National Key Research and Development Program Project of China, 2022YFF1100102. Cross‐Innovation Open Project of Food Flavor and Health, Beijing Technology & Business University, FFHCI‐2025071.

## Disclosure

The authors have nothing to report.

## Supporting information


**Table S1:** Effects of ALA on organ weight of HFD‐fed mice.
**Figure S1:** The effects of ALA and LPS on the viability of Caco‐2 cells. (a) The effect of ALA on Caco‐2 cell viability, (b) The effect of LPS on Caco‐2 cell viability (different letters in the figure indicate significant differences, *p* ≤ 0.05, and there is no significant difference with the same letter).

## Data Availability

All data included in this study are available upon request by contact with the corresponding author.
