# Supplementary material for: α‐Linolenic Acid Alleviated Intestinal Inflammation and Dyshomeostasis Induced by Obesity in Mice
Source: Food Sci Nutr. 2026 Mar 23;14(3):e71680. doi: 10.1002/fsn3.71680 (PMC13093600; doi:10.1002/fsn3.71680)
Supplement: Supplementary file 1 — Table S1: Effects of ALA on organ weight of HFD‐fed mice. Figure S1: The effects of ALA and LPS on the viability of Caco‐2 cells. (a) The effect of ALA on Caco‐2 cell viability, (b) The effect of LPS on Caco‐2 cell viability (different letters in the figure indicate significant differences, p ≤ 0.05, and there is no significant difference with the same letter). [file FSN3-14-e71680-s001.docx]

**Supplementary:**

Table S1 Effects of ALA on organ weight of HFD-fed mice

|  | Con | HFD | L-LA | M-LA | H-LA |
| --- | --- | --- | --- | --- | --- |
| heart（g） | 0.158±0.013^a^ | 0.203±0.034^b^ | 0.183±0.036^ab^ | 0.151±0.020^a^ | 0.184±0.045^ab^ |
| liver（g） | 1.016±0.094^a^ | 1.201±0.108^b^ | 0.938±0.100^ac^ | 0.869±0.082^c^ | 0.924±0.098^ac^ |
| spleen（g） | 0.064±0.019^a^ | 0.088±0.013^b^ | 0.088±0.013^b^ | 0.073±0.029^ab^ | 0.089±0.024^ab^ |
| kidney（g） | 0.386±0.023^a^ | 0.444±0.062^b^ | 0.386±0.053^ab^ | 0.366±0.061^a^ | 0.394±0.048^ab^ |
| Epididymal fat（g） | 0.749±0.199^a^ | 2.453±0.596^b^ | 1.958±0.578^bc^ | 1.628±0.251^c^ | 1.500±0.537^c^ |

Note: Values are expressed as mean ± SEM; Different letters above the mean indicate significant differences (P ≤ 0.05), while the same letter indicates no significant differences.

(a) (b)

Fig. S1 The effects of ALA and LPS on the viability of Caco-2 cells. (a) The effect of ALA on Caco-2 cell viability, (b) The effect of LPS on Caco-2 cell viability (different letters in the figure indicate significant differences, P ≤ 0.05, and there is no significa nt difference with the same letter).
